# A Study of Patients with Primary Mediastinal Germ Cell Tumors Treated Using Multimodal Therapy

**DOI:** 10.1155/2017/1404610

**Published:** 2017-05-28

**Authors:** Yutaro Tanaka, Takehiko Okamura, Takashi Nagai, Daichi Kobayashi, Takahiro Kobayashi, Hidetoshi Akita, Yoshinobu Moritoki, Takahiro Yasui

**Affiliations:** ^1^Department of Urology, Anjo Kosei Hospital, Anjo, Japan; ^2^Department of Nephro-Urology, Nagoya City University Graduate School of Medical Sciences, Nagoya, Japan

## Abstract

**Objectives:**

Primary mediastinal germ cell tumors (PMGCTs) are rare, which often makes them difficult to treat. Herein, we examined patients with PMGCTs who underwent multimodal treatment.

**Methods:**

We examined 6 patients (median age: 25 years, range: 19–27 years) with PMGCTs who underwent multimodal treatment between April 2001 and March 2015. Three patients had seminomas, 2 patients had yolk sac tumors, and 1 patient had choriocarcinoma. The median observation period was 32.5 months (range: 8–84 months).

**Results:**

Three of the 6 patients received initial operation followed by 3-4 courses of chemotherapy (bleomycin, etoposide, and cisplatin (BEP) or etoposide and cisplatin (EP)). One patient developed multiple lung metastases 17 months after surgery; received salvage chemotherapy with vinblastine, ifosfamide, and cisplatin; and achieved complete remission. The remaining 3 patients received initial BEP and EP chemotherapy. Multiple lung metastases and supraclavicular lymph node metastases were detected in 2 of these patients at the initial diagnosis. The patients underwent resections to remove residual tumor after treatment, and no viable tumor cells were found.

**Conclusions:**

Reliable diagnosis and immediate multimodal treatments are necessary for patients with PMGCTs. The 6 patients treated in our hospital have never experienced recurrence after the multimodal treatment.

## 1. Introduction

Primary mediastinal germ cell tumor (PMGCT) is a rare cancer that accounts for less than 5% of germ cell malignancies and only 1–4% of all mediastinal tumors [1, 2]. The histopathological characteristics of PMGCTs are similar to those of gonadal germ cell tumors (GCTs). However, they have different biological behaviors, clinical characteristics, and overall prognoses [3]. Patients with pure seminomas in the mediastinum reportedly have an almost 90% chance of long-term cure, but those with nonseminoma tumors have only about a 45% chance of cure [4].

Much attention is being paid to therapeutic strategies. Chemotherapy for PMGCT has already become a standard practice since the introduction of cisplatin-based combination chemotherapy. With standard chemotherapy consisting of 4 courses of bleomycin, etoposide, and cisplatin (BEP), men with nonseminoma mediastinal tumors have experienced better prognoses, although not better than patients with mediastinal seminomas or primary tumors of the testis [5].

Single chemotherapeutic agents have a limited ability to treat PMGCT. Surgical treatment in addition to chemotherapy can lead to a better prognosis. Recently, cisplatin-based chemotherapy regimens were reported to provide a maximum effect when followed by surgical consolidation, resulting in long-term progression-free survival and overall survival [6]. However, the clinical implications of resection in the multimodality treatment of PMGCTs are not yet clear. Owing to the rarity of this tumor, the lack of consensus on the treatment strategies, and the different conditions at each institution, there have not been satisfactory outcomes for patients with PMGCT until now.

In this study, we examined patients in our hospital whose PMGCTs were treated using multimodal treatment.

## 2. Methods

Between April 2001 and March 2015, 6 patients with PMGCTs underwent multimodal treatment at the Anjo Kosei Hospital.  Table 1 shows the characteristic features of the 6 patients. All 6 patients were men, with a median age of 25 years (range: 19–27 years) and median body mass index of 22.1 kg/m^2^ (range: 16.6–41.8 kg/m^2^).

Five patients had symptoms at the time of diagnosis, with chest pain and cough in 2 patients each and dyspnea in 1 patient; 1 patient was asymptomatic. All patients underwent standard chest radiography and computed tomography (CT). There were bulky masses located in the anterior mediastinum in all patients. In 2 of the 6 patients, metastases were found at the initial diagnosis, with 1 patient having multiple lung metastases and the other having supraclavicular lymph node metastases. There was no clinical or imaging detection of testicular cancer.

Histopathological diagnoses were assessed for 3 patients using fine needle biopsy-guided ultrasonography or CT. Those histopathological evaluations resulted in diagnoses in 2 cases: 1 seminoma and 1 yolk sac tumor. One case could not be diagnosed by biopsy and was diagnosed via initial resection as a seminoma. The remaining 3 patients did not undergo a biopsy, and their tumors were diagnosed using surgical specimens or serum tumor markers, such as human chorionic gonadotropin (hCG), alpha-fetoprotein (AFP), and lactate dehydrogenase (LDH). There were 3 patients with seminoma and 3 patients with nonseminoma tumors (2 yolk sac tumors and 1 choriocarcinoma).

All patients were treated with a combination of chemotherapy and surgery. The patients received neoadjuvant chemotherapy when they were diagnosed with PMGCT by biopsy or serum tumor markers. If, after discussion with a thoracic surgeon, the tumor was deemed anatomically radically resectable, we performed an initial surgical treatment, even if the patient had already been diagnosed with PMGCT. When the disease was difficult to diagnose by biopsy or serum tumor markers, patients underwent surgical treatment before starting chemotherapy.

Four of the 6 patients were diagnosed before treatment. Among the 4 diagnosed patients, 3 were initially treated with chemotherapy. In the remaining patient, thoracic radical resection was possible, and thus surgery was the initial treatment. In the 2 patients who could not be diagnosed, surgery was performed prior to starting chemotherapy. Initial chemotherapy consisted of either a combination of cisplatin, bleomycin, and etoposide (BEP) or a combination of cisplatin and etoposide (EP). We began with a BEP regimen; however, we switched to an EP regimen owing to some patients having adverse reactions to bleomycin, including lung damage or fever. BEP chemotherapy consisted of intravenous cisplatin (20 mg/m^2^) and intravenous etoposide (100 mg/m^2^) on days 1–5 and intravenous bleomycin (30 mg total) on days 1, 8, and 15. EP chemotherapy consisted of intravenous cisplatin (20 mg/m^2^) and intravenous etoposide (100 mg/m^2^) on days 1–5. For second-line chemotherapy and salvage chemotherapy, we administered vinblastine, ifosfamide, and cisplatin (VeIP), with intravenous ifosfamide (1200 mg/m^2^) and intravenous cisplatin (20 mg/m^2^) administered on days 1–5 and vinblastine (0.11 mg/kg) administered on days 1 and 2. The median observation period was 32.5 months (range: 8–84 months).

All patients gave their informed consent, and the protocol for this research project has been approved by a suitably constituted ethics committee of Anjo Kosei Hospital. This work also conforms to the provisions of the Declaration of Helsinki (as revised in Fortaleza, Brazil, October 2013).

## 3. Results

Table 2 shows the outcomes for all 6 cases.

### 3.1. Three Patients Underwent Initial Surgical Treatment

There were no metastases in any of these patients at the initial diagnosis. In 2 of the seminoma cases, it was difficult to determine an exact diagnosis by biopsy or serum tumor markers, and thus initial surgical treatment was performed. These patients received 3-4 courses of postoperative BEP and EP chemotherapy. One patient with seminoma developed multiple lung metastases 17 months after treatment and received salvage chemotherapy with 2 courses of VeIP. The patient then achieved complete remission (CR). In 1 case of yolk sac tumor, on the basis of the preoperative biopsy and high levels of AFP, we performed initial surgical treatment because the tumor was anatomically thoracically resectable. After the operation, the patient received 4 courses of postoperative BEP and EP chemotherapy. The serum tumor markers gradually decreased and normalized.

### 3.2. Three Patients Underwent Initial Chemotherapy

Three patients were diagnosed by biopsy or elevated serum tumor markers; each had a different pathological diagnosis (seminoma, choriocarcinoma, and yolk sac tumor). In patients with diagnoses of seminoma and choriocarcinoma, supraclavicular lymph node metastases and multiple lung metastases were detected, respectively, at the initial diagnosis. All 3 patients received 3-4 courses of neoadjuvant chemotherapy, either BEP or EP after intolerable BEP (Table 2). In the patient with seminoma, the right supraclavicular lymphadenopathy was not reduced, even though 4 courses of BEP and EP were administered. The patient received second-line chemotherapy with 2 courses of VeIP and achieved CR. Both the patient with choriocarcinoma and the patient with the yolk sac tumor received BEP or EP until their serum tumor markers normalized. Finally, resections were performed to remove residual tumor after treatment, and no viable cells were found in any of these 3 cases.

All of the patients are alive and recurrence-free after a median follow-up of 32.5 months, regardless of their primary treatment method.

Representative examples of initial chemotherapy treatment and initial surgical treatment are further detailed below.

#### 3.2.1. Case Presentation 1: Initial Chemotherapy

A previously healthy 22-year-old Japanese man without a medical or surgical history presented to Anjo Kosei Hospital with complaints of fever and dyspnea. Physical examination revealed a temperature of 37.8°C and tachycardia. CT scans revealed a 14.4 × 9.8 × 10.0 cm mass in the anterior mediastinum with compression of the heart. Pertinent laboratory values included an AFP level of 31,536 ng/mL and an LDH level of 604 IU/L. His hCG level was within the normal range. Physical examination of the testes showed no neoplastic parenchymal involvement. A needle core biopsy revealed a malignant germ cell tumor, and immunohistochemical staining was positive for AFP. From these data, the patient was finally diagnosed with a yolk sac tumor.

**Figure 1:**
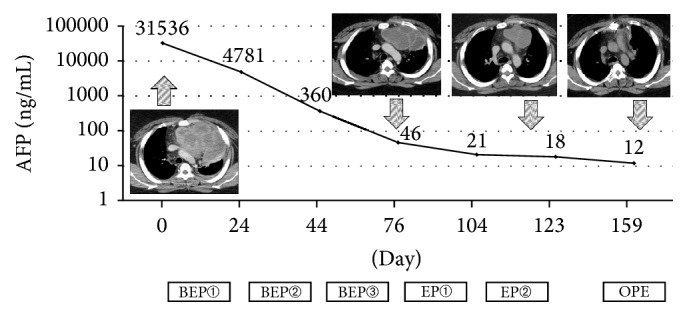
Case 1: a 22-year-old man received initial chemotherapy treatment. AFP: alpha-fetoprotein; BEP: bleomycin, etoposide, and cisplatin; EP: etoposide and cisplatin; OPE: operation.

The patient received combination chemotherapy consisting of BEP every 3 weeks for a total of 3 cycles. After that, an additional 2 cycles of EP chemotherapy were administered in order to normalize his serum tumor marker levels. His AFP level decreased to 46 ng/mL after 3 cycles of BEP and further normalized after 2 cycles of EP. After 5 courses of preoperative chemotherapy, resection of the tumor was performed, resulting in a complete pathologic response (Figure 1). The patient is alive and in CR without any evidence of recurrence.

#### 3.2.2. Case Presentation 2: Initial Surgery

**Figure 2:**
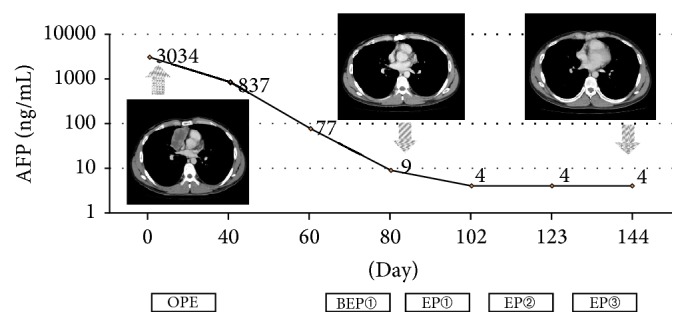
Case 2: a 25-year-old man received initial surgical treatment. AFP: alpha-fetoprotein; BEP: bleomycin, etoposide, and cisplatin; EP: etoposide and cisplatin; OPE: operation.

A previously healthy 25-year-old Japanese man was admitted to our hospital with complaints of chest pain. His history was unremarkable. Physical examination did not reveal any significant findings. A CT scan revealed a 7.6 × 6.5 × 3.5 cm mass in the anterior mediastinum and no metastases at the initial diagnosis. His laboratory values included an AFP level of 3,034 ng/mL and an hCG level of 20.6 ng/mL. Ultrasonography of the testes revealed a uniform pattern without any findings suggestive of malignancy. Needle core biopsy was not performed. Based on suspicion for PMGCT, the patient was referred for thoracic surgery. The tumor was histopathologically diagnosed as a yolk sac tumor. After the operation, the patient received 4 total courses of BEP and EP chemotherapy. Upon completion of the chemotherapy, his serum tumor marker levels normalized (Figure 2). The patient is alive and in CR without any evidence of recurrence after 3 years.

## 4. Discussion

Most GCTs occur in the gonads, but PMGCTs occur in the mediastinum. Although general histologic and serologic characteristics of PMGCTs are similar to those of testicular GCTs, patients with PMGCTs have poorer prognoses than those with testicular GCTs [7, 8]. However, the reasons for the differences in clinical characteristics and behaviors of PMGCT are still unknown.

PMGCTs are generally divided into two groups: seminomas and nonseminoma tumors. Nonseminoma tumors include yolk sac tumors, embryonal carcinoma, choriocarcinoma, mature and/or immature teratoma, and mixed tumors. Of all PMGCTs, 37% are seminomas, 16% are nonseminoma tumors (not including teratomas), 27% are mature teratomas, 16% are immature teratomas, and 4% are mixed tumors [9]. Among nonseminomatous PMGCTs, yolk sac tumors are the most common, accounting for 12% of all PMGCTs [10]. At our institution, 3 of the 6 cases (50%) were seminomas, and 2 of the remaining 3 nonseminoma tumors were yolk sac tumors.

Treatment strategies for PMGCTs are extremely important. Patients with seminomas have good prognoses with chemotherapy and surgery or radiation in most cases because of the high sensitivity of the tumor to radiation [3, 11, 12]. However, this therapy is not effective for nonseminoma tumors. Therefore, the current consensus is that initial systemic chemotherapy should be followed by aggressive complete resection of all macroscopic tumors [12–16]. However, patients with nonseminoma tumors reportedly have a 5-year overall survival rate of only 45%, compared with an almost 90% rate of long-term cure for patients with seminoma, which is not satisfactory [4, 16].

Several studies indicate that histology, location, distant metastases, number of metastatic sites, treatment strategies, pathological evidence of persistent viable cells in the resected specimen, and serum tumor marker levels before or after operation are independent prognostic factors for patients with PMGCTs [17–19]. All 6 patients from our series remain recurrence-free. Our study likely had these satisfactory outcomes for three reasons. First, we cooperated well with other departments, allowing us to provide individualized treatment modalities, and all patients received combined surgery and chemotherapy. Second, in cases treated with initial surgery, we were able to perform radical resections. Finally, in cases treated with initial chemotherapy administration, we achieved normalization of serum tumor marker levels with no viable cells in the resected specimen.

Liu et al. demonstrated that chemotherapy combined with surgery or radiotherapy resulted in the longest survival time for patients with PMGCT, compared with monotherapy, whether chemotherapy, surgery, or radiotherapy. The greater and prolonged toxicities of the three treatments and the inability to eliminate residual tumor with monotherapy are the reasons for this difference [20]. As in most series, patients treated with initial chemotherapy followed by surgery had a superior outcome to those treated with initial surgery [16]. On the other hand, thoracotomy should first be performed for radical resection followed by postoperative chemotherapy if it is anatomically feasible, such as for small and resectable masses without any invasion [20]. In our institution, we usually treat patients with PMGCTs initially with chemotherapy, but, in some cases, we may perform surgery initially if the tumor can be radically resected. However, thoracic surgery requires different skilled treatment strategies in each institution. There is a greater need for interdepartmental collaboration in the treatment of PMGCT, as combining chemotherapeutics and surgery provides the best outcome in many cases. The best treatment strategies for each patient should be chosen after discussing the strategy with other departments. We have a substantial thoracic surgery practice, which led to good results in our patients.

Serum tumor marker levels after chemotherapy, timing of surgical treatment, and evidence of persistent viable cells in resected specimens are important prognostic factors for PMGCT. Wright et al. reported that normalization of serum tumor marker levels before surgical treatment is a significant favorable prognostic factor. Therefore, patients with elevated tumor marker levels after first-line chemotherapy should receive second-line chemotherapy before surgery, as in our cases [21]. However, Kesler et al. reported that patients should undergo resection after first-line chemotherapy, regardless of the high levels of serum tumor markers [18]. They emphasized that second-line chemotherapy for patients with nonseminomatous PMGCTs resulted in only an 11% long-term disease-free survival rate [22]. Fortunately, in our series, the serum tumor marker levels for all patients had normalized before operation. Additionally, no viable cells were found pathologically in the resected specimens. In this study, preoperative normal tumor marker levels may have been the most important prognostic factor.

In conclusion, we have successfully treated 6 patients with PMGCTs. As a result, we suggest that all patients should receive both surgery and chemotherapy to achieve normalization of serum tumor marker levels and eliminate viable cells in the residual tumor. The initial therapy, whether surgical treatment or chemotherapy, is influenced by many factors, such as patient status, tumor size and invasion, skill and experience of the oncology center, and especially the expertise of the thoracic surgical oncologists. Therefore, it is difficult to standardize treatment options. However, multimodal treatment has become the standard treatment for PMGCT. In order to achieve successful outcomes, we should communicate with other departments to treat the patient as an entire medical team. We use a multidisciplinary team system in our hospital, which likely led to these satisfactory results.

## Figures and Tables

**Table 1:** Characteristics of the six patients with primary mediastinal germ cell tumors.

Characteristic	*n* = 6
Median age (years)	25 (19–27)
Median body mass index (kg/m^2^)	22.1 (16.6–41.8)
Sex (male/female)	6/0
Median follow-up (months)	32.5 (8–84)
Histopathology	
Pure seminoma	3 (50%)
Yolk sac	2 (33%)
Choriocarcinoma	1 (17%)
Extramediastinal metastases	
Lung	1 (17%)
Supraclavicular lymph nodes	1 (17%)

**Table 2:** Clinical characteristics, treatments, and outcomes of the six patients with primary mediastinal germ cell tumors.

	Age	Sex	BMI	Metastases	Histopathology	Preceding chemotherapy (courses)	Preoperative chemotherapy (courses)	Recurrence	Salvage chemotherapy (courses)	Follow-up (months)
*Preceding surgery*										
Case 1	27	Male	21.0	No	Seminoma	—	BEP (1)/EP (2)	Lung metastasis	VeIP (2)	36
Case 2	25	Male	23.1	No	Yolk sac	—	BEP (1)/EP (3)	No	No	36
Case 3	19	Male	16.6	No	Seminoma	—	BEP (1)/EP (2)	No	No	29
*Preceding chemotherapy*										
Case 4	27	Male	19.1	Supraclavicular lymph node	Seminoma	BEP (1)/EP (3)/VeIP (2)	—	No	No	84
Case 5	22	Male	41.8	No	Yolk sac	BEP (3)/EP (2)	—	No	No	16
Case 6	25	Male	24.9	Lung	Choriocarcinoma	BEP (3)/EP (1)	—	No	No	8

BEP: bleomycin, etoposide, and cisplatin; BMI: body mass index; EP: etoposide and cisplatin; VeIP: vinblastine, ifosfamide, and cisplatin.
